# PERSONALITY AND PLASMA BIOMARKERS OF REACTIVE ASTROGLIOSIS (GFAP) AND NEURONAL INJURY (NFL)

**DOI:** 10.1093/geroni/igad104.1398

**Published:** 2023-12-21

**Authors:** Antonio Terracciano, Keenan Walker, Yang An, Martina Luchetti, Abhay R Moghekar, Angelina Sutin, Luigi Ferrucci, Susan Resnick

**Affiliations:** Florida State University, Tallahassee, Florida, United States; National Institute on Aging, National Institutes of Health, Baltimore, Maryland, United States; National Institute on Aging, National Institutes of Health, Baltimore, Maryland, United States; Florida State University, College of Medicine, Tallahassee, Florida, United States; Johns Hopkins University, Baltimore, Maryland, United States; Florida State University, College of Medicine, Tallahassee, Florida, United States; National Institute on Aging, National Institutes of Health, Baltimore, Maryland, United States; National Institute on Aging, National Institutes of Health, Baltimore, Maryland, United States

## Abstract

Personality traits are associated with cognitive functioning and risk of Alzheimer’s disease and related dementias. To understand the biological mechanisms of these associations, it is essential to examine the amyloid (A), tau (T), and neurodegeneration (N) components of the ATN framework. A recent meta-analysis found that neuroticism and conscientiousness are associated with amyloid and tau. However, less is known on whether personality traits are associated with markers of neurodegeneration. This study examines whether personality traits are concurrently related to plasma glial fibrillary acidic protein (GFAP), a marker of reactive astrogliosis, and neurofilament light (NfL), a marker of neuronal injury. Cognitively unimpaired participants from the Baltimore Longitudinal Study on Aging (N=786; age: 22-95) were assayed for plasma GFAP and NfL and completed the Revised NEO Personality Inventory (NEO-PI-R), which measures five domains and 30 facets of personality. Neuroticism (particularly vulnerability to stress, anxiety, and depression facets) was associated with higher GFAP and NfL. Conscientiousness was associated with lower GFAP. Extraversion (particularly positive emotions, assertiveness, and activity facets) was related to lower GFAP and NfL. These associations were independent of demographic, behavioral, and health covariates and not moderated by age, sex, or APOE genotype. The personality correlates of astrogliosis and neuronal injury tend to be similar, are found in individuals without cognitive impairment, and point to potential neurobiological underpinnings of the association between personality traits and neurodegenerative diseases.

